# Surveillance of pyrazinamide susceptibility among multidrug-resistant *Mycobacterium tuberculosis *isolates from Siriraj Hospital, Thailand

**DOI:** 10.1186/1471-2180-10-223

**Published:** 2010-08-20

**Authors:** Jirarut Jonmalung, Therdsak Prammananan, Manoon Leechawengwongs, Angkana Chaiprasert

**Affiliations:** 1 Department of Microbiology, Faculty of Medicine Siriraj Hospital, Mahidol University, Bangkok, 10700, Thailand; 2 National Center for Genetic Engineering and Biotechnology, National Science and Technology, Development Agency, Ministry of Science and Technology, Pathumthani 12120, Thailand; 3 Vichaiyut Hospital, Setsiri Road, Bangkok 10400, Thailand; 4 Drug-Resistant Tuberculosis Research Fund, Siriraj Foundation, Bangkok 10700, Thailand

## Abstract

**Background:**

Susceptibility testing of pyrazinamide (PZA) against *Mycobacterium tuberculosis *is difficult to perform because the acidity of culture medium that is required for drug activity also inhibits the growth of bacteria. In Thailand, very limited information has been generated on PZA resistance, particularly among multidrug-resistant tuberculosis (MDR-TB) isolated from Thailand. Only two studies on PZA susceptibility among Thai *M. tuberculosis *strains have been reported; one used a pyrazinamidase assay, and the other used the BACTEC 460 TB for PZA susceptibility testing. In this study, we determined the percentage of strains possessing pyrazinamide resistance among pan-susceptible *M. tuberculosis *and MDR-TB isolates by using the pyrazinamidase assay, BACTEC MGIT 960 PZA method and *pncA *sequencing, and assessed the correlation in the data generated using these methods. The type and frequency of mutations in *pncA *were also determined.

**Results:**

Overall, 150 *M. tuberculosis *isolates, consisting of 50 susceptible and 100 MDR-TB isolates, were tested for PZA susceptibility by BACTEC MGIT 960 PZA, the pyrazinamidase assay and *pncA *sequencing. The study indicated PZA resistance in 6% and 49% of susceptible and MDR-TB isolates, respectively. In comparison to the BACTEC MGIT 960 PZA, the PZase assay showed 65.4% sensitivity and 100% specificity, whereas *pncA *sequencing showed 75% sensitivity and 89.8% specificity. Twenty-four mutation types were found in this study, with the most frequent mutation (16%) being His71Asp. Of these mutations, eight have not been previously described. The Ile31Ser and Ile31Thr mutations were found both in PZA susceptible and resistant isolates, suggesting that mutation of this codon might not play a role on PZA resistance.

**Conclusions:**

Our findings suggest that phenotypic susceptibility testing is still essential for the detection of PZA resistance, especially for MDR-TB isolates. Some mutations were not associated with resistance and could lead to misinterpretation of the genotypic methods. This information could be helpful for clinicians in managing tuberculosis patients and frequencies, and the types of *pncA *mutations should offer baseline information on PZA resistance.

## Background

Tuberculosis (TB) is a public health problem caused by *Mycobacterium tuberculosis*. Thailand was ranked 18^th ^among high-burden countries, with 91,000 cases per year and new cases of MDR-TB (resistance to at least isoniazid and rifampicin) of approximately 1.7% [1]. Tuberculosis infection is increasing in human immunodeficiency virus (HIV) co-infected patients, affecting the TB control program as about one-third of Thai HIV/AIDS patients present with active TB [2-5]. The standard regimen for the treatment of TB consists of 2 months of intensive treatment with isoniazid, rifampicin, ethambutol, and pyrazinamide (H, R, E, and Z), followed by 4 months of maintenance treatment with isoniazid and rifampicin (H and R). Whereas other first-line drugs do not reveal any problem for susceptibility testing, this is not true for pyrazinamide, as it is active against tubercle bacilli only at an acidic pH (e.g., pH 5.5), resulting in that it cannot use conventional culture medium for susceptibility testing [6].

Pyrazinamide (PZA, Z) is a prodrug that requires conversion to the active form, pyrazinoic acid (POA), by mycobacterial pyrazinamidase (PZase) [7]. The exact target of POA is unknown. It has been suggested that the accumulation of POA in acidic conditions (from lactic acid produced by inflammation cells) leads to acidification of the cytoplasm and subsequent cell damage [7,8]. Mycobacterial pyrazinamidase is encoded by *pncA*, and mutations in this gene have been demonstrated as the major mechanism of PZA resistance [9]. Several mutations, including missense, insertion, deletion and nonsense mutations, have been reported and located in both the putative promoter and coding regions of *pncA *[10]. PZA-resistant *M. tuberculosis *strains are usually correlated with defective PZase activity, but some PZA resistant strains have been reported to contain wild-type *pncA *and to maintain PZase activity [11-14], suggesting that other unknown resistance mechanisms could be responsible for the resistance phenotype.

PZA susceptibility testing is difficult because the acidity of culture medium needed for drug activity also restricts the growth of *M. tuberculosis*. The use of large inoculum sizes results in the release of NH_3_, leading to increased pH and inactivated PZA [7]. The BACTEC 460 TB radiometric method has been validated and developed as the reference method for PZA susceptibility testing [15]. Recently, PZA susceptibility testing has been performed by the nonradiometric, fully automated, continuous-monitoring MGIT 960 system (Becton Dickinson), which produced a rapid and reliable result [16,17]. Many studies revealed a good correlation between loss of PZase activity and resistance to PZA [18-22]. Thus, the detection of PZase activity has been used for PZA susceptibility testing. Nevertheless, various levels of sensitivity (79-96%) of the PZase assay for PZA susceptibility testing have been reported [20-22]. In Thailand, only two studies on PZA susceptibility among Thai *M. tuberculosis *strains have been reported, and the results revealed that the initial PZA resistance was 5.95% and 7.8% when detected by the PZase assay [18] and by BACTEC 460 TB [23], respectively. In this study, we determined the percentage of strains that exhibited pyrazinamide resistance among pan-susceptible *M. tuberculosis *and MDR-TB isolates by using the pyrazinamidase assay, BACTEC MGIT 960 PZA method and *pncA *sequencing, and we evaluated the correlation of the results obtained with these methods. *pncA *mutation type and frequency were also evaluated.

## Methods

### Mycobacterial isolates

During 2005-2007, there were 4,536 *M. tuberculosis *isolates from 7,807 sputum samples sending from all parts of Thailand (118 hospitals and 43 of 76 provinces) to the Molecular Mycology and Mycobacteriology Laboratory, Drug-Resistant Tuberculosis Research Fund, Department of Microbiology, Faculty of Medicine Siriraj Hospital, Mahidol University. Of these, 220 and 4,316 isolates were identified as MDR-TB and non MDR-TB, including pan-susceptible isolates respectively. One hundred and fifty *M. tuberculosis *clinical isolates, consisting of 50 pan-susceptible isolates (susceptible to isoniazid, rifampicin, ethambutol, and streptomycin) and 100 isolates of MDR-TB, were selected based on their ability to re-cultivate from stock cultures and availability of demographic data. The MDR-TB isolates contain 17, 13, 26 and 44 isolates resisted to isoniazid and rifampicin, to isoniazid, rifampicin and streptomycin, to isoniazid, rifampicin and ethambutol and to all four drugs respectively. These isolates were identified to species using the in-house one-tube multiplex PCR [24], and antimicrobial susceptibility testing to isoniazid, rifampicin, ethambutol and streptomycim was performed by the standard proportion method on M7H10 agar as recommended by the CDC [25] and NCCLS [15]. Each isolate obtained from individual patient. The patients include new and previously treated patients with the age ranged from 5 to 81 years and comprised of 65% male and 35% female. Only 21% were known human immunodeficiency virus (HIV) status. Among these, 52% were HIV-positive.

### PZA susceptibility testing

Pyrazinamide susceptibility testing was performed using the BACTEC MGIT 960 PZA system (Becton Dickinson) as recommended by the manufacturer. The medium used was modified Middlebrook 7H9 broth (pH 5.9) containing 100 μg/ml PZA. *Mycobacterium bovis *BCG ATCC 34540 and *Mycobacterium tuberculosis *H37Rv ATCC 27294 were used as pyrazinamide resistant and susceptible controls, respectively. The control strains were included in all test sets.

### Pyrazinamidase assay

Pyrazinamidase activity was determined by Wayne's method [26]. This method is based on the detection of POA, which forms a compound with ferrous ammonium sulphate to produce a brownish or pink colour. Briefly, a heavy loopful of *M. tuberculosis *colonies was obtained from cultures that were actively growing in LJ medium and inoculated onto the surfaces of two agar butt tubes, each containing 5 ml of Wayne's medium supplemented with 100 μg/ml of PZA (Sigma-Aldrich, USA). The tubes were incubated at 37°C. Four days after incubation, 1 ml of freshly prepared 1% ferrous ammonium sulphate was added to the first tube. The tube was left at room temperature for 30 minutes and examined. The assay was positive if a pink or brownish band was present on the surface of the agar. If the test was negative, the test was repeated with a second tube and examined after 7 days of incubation. The results were blindly read by two independent observers. *M. bovis *BCG and *M. tuberculosis *H37Rv were used as negative and positive controls, respectively.

### DNA extraction

Mycobacterial DNAs were extracted by the boiling method [27]. Briefly, one loopful of *M. tuberculosis *colonies obtained from LJ medium was suspended in 200 μl of TE buffer (10 mM Tris-HCl, 1 mM EDTA, pH 8.0) and boiled for 20 minutes. The supernatant was collected by centrifugation at 12,000 rpm for 5 min and used as the DNA template for amplification.

### Amplification and sequencing of the amplified *pncA *gene

The *pncA *forward primer, pncAF1, (5'-GCGGCGTCATGGACCCTATATC-3') was located 82 bp upstream of the start codon, and the reverse primer, pncAR1, (5'-CTTGCGGCGAGCG CTCCA -3') was located 54 bp downstream of the stop codon of *M. tuberculosis pncA *(Rv2043c). The expected size of the PCR products was 696 bp. PCR was performed in a total volume of 50 μl, and the PCR reaction mixture consisted of 0.25 mM dNTP (Fermentas, CA, USA), 10 mM Tris-HCl (pH 8.3), 50 mM KCl, 2.0 mM MgCl_2_, 20 pmol of each primer, 1 unit of *Taq *DNA polymerase (Fermentas, CA, USA) and 5 μl of crude DNA. The PCR reactions were performed under the following conditions: initial denaturation at 94°C for 5 min; 40 cycles of denaturation at 94°C for 1 min, annealing at 62°C for 1 min and extension at 72°C for 1 min; and 1 final cycle of extension at 72°C for 10 min. PCR products were analysed by 1% agarose gel electrophoresis, and subsequently they were purified using the Gel Extraction Kit (Qiagen, Germany). Finally, purified DNAs were directly sequenced with the ABI PRISM 3730XL Analyzer, (Applied Biosystems, Foster City, USA) using the pncAF1 and pncAR1 primers as sequencing primers. The obtained sequences were compared with the sequence of *M. tuberculosis *H37Rv *pncA *(Accession no. NC_000962) by using the blastn program http://blast.ncbi.nlm.nih.gov/Blast.cgi.

## Results

### Pyrazinamide susceptibility testing by the phenotypic method

MGIT 960 susceptibility testing demonstrated that 52 (34.6%) of 150 isolates were phenotypically resistant to PZA. More specifically, 3 (6%) of 50 pan-susceptible *M. tuberculosis *isolates were resistant to PZA, whereas 49 (49%) of 100 MDR-TB isolates were PZA-resistant, as summarised in Table 1.

**Table 1:** Comparison of *pncA *sequencing, the pyrazinamidase assay, and the MGIT 960 system for PZA susceptibility testing.

*M. tb *strains (total no. of isolates)	MGIT (S) PZase (pos) *pncA (wt)*	MGIT (S) PZase (pos) *pncA (mut)*	MGIT (R) PZase (neg) *pncA (mut)*	MGIT (R) PZase (pos) *pncA (wt)*	MGIT (R) PZase (pos) *pncA (mut)*
Susceptible (50)	46	1	-	2	1
MDR-TB (100)	42	9	34	11	4

S; susceptible, R; resistant, PZase; pyrazinamidase assay, MGIT; BACTEC MGIT 960 method, pos; positive, neg; negative, wt; wild-type, mut; mutant

### Correlation of PZA susceptibility testing and the pyrazinamidase assay

Pyrazinamidase activity was detected in all pan-susceptible isolates and in 3 PZA-resistant isolates. Among the 100 MDR-TB isolates, 85 provided concordant results between the two methods; 51 isolates with phenotypic susceptibility to PZA had PZase activity, whereas PZase activity could not be detected in 34 PZA-resistant isolates. However, 15 MDR-TB isolates with PZA-resistant phenotypes had PZase activity (Table 1). Compared to the BACTEC MGIT 960 PZA system, the PZase assay showed 65.4% sensitivity and 100% specificity.

### Correlation of PZA susceptibility, pyrazinamidase assay and mutations in *pncA*

Susceptibility testing by BACTEC MGIT 960 PZA revealed 98 PZA-susceptible isolates with positive PZase activity. Of these, 88 isolates had no mutations in *pncA*, whereas 10 isolates harboured mutations at nucleotide 92 (T → G/C), causing an amino acid change from isoleucine to serine or threonine, respectively, at codon 31. Thirty-two of the PZA-resistant isolates without PZase activity contained mutations in *pncA*, with 18 types of nucleotide substitutions in the coding region, 2 mutational types in the putative promoter region, 2 nucleotide insertions, and one nonsense mutation, as summarised in Table 2. Interestingly, there were two PZA-resistant isolates with negative PZase activity that were mutated at codon 31 (Ile→Ser), a mutant that was also found in PZA-susceptible isolates. In contrast, five PZA-resistant isolates that had Ile31Ser or Ile31Thr mutations possessed PZase activity (Table 2). Moreover, there were 13 (26.5%) isolates with wild-type *pncA *and PZase activity but possessed resistant phenotypes. Thus, the sensitivity and specificity of *pncA *sequencing were 75% and 89.8% respectively, when compared with the BACTEC MGIT 960 PZA.

**Table 2:** Results of *pncA *gene sequencing of 150 *M. tuberculosis *clinical isolates.

*M. tuberculosis *strains (no. of isolates)	MGIT 960	PZase assay	*pncA *mutation	
			
			Nucleotide change	Amino acid change
Susceptible (46)	S	+	wild-type	no
Susceptible (1)	S	+	T92G	Ile31Ser
Susceptible (2)	R	+	wild-type	wild-type
Susceptible (1)	R	+	T92C	Ile31Thr

MDR-TB (42)	S	+	wild-type	wild-type
MDR-TB (9)	S	+	T92C	Ile31Thr
MDR-TB (34)	R	-	A(-11)G (1)	no
			A(-11)C (1)	no
			T56G (1)	Leu19Arg
			T80C (1)	Leu27Pro
			T92G (2)	Ile31Ser
			T104C (1)	Leu35Pro
			T134C (1)	Val45Ala
			G136T (1)	Ala46Ser
			T199C (1)	Ser67Pro
			C211G (8)	His71Asp
			G215A (1)	Cys72Tyr
			G222C (1)	Gly74Arg
			G289A (3)	Gly97Ser
			C312G (2)	Ser104Arg
			G364A (1)	Gly122Ser
			G373T (1)	Val125Phe
			G379T (1)	Glu 127 Stop
			G insertion b/w 411-412 (1)	
			T416G (1)	Val 139 Gly
			C425T (1)	Thr 142 Met
			G436A (1)	Ala 146 Thr
			C520T (1)	Thr 174 Ile
			GG insertion b/w 520-521 (1)	
MDR-TB (11)	R	+	wild-type	no
MDR-TB (4)	R	+	T92C (3)	Ile31Thr
			T92G (1)	Ile31Ser

## Discussion

Several studies have reported that the prevalence of PZA resistance ranges from 36% to 54% [14,28,29]. In Thailand, there is little information on PZA susceptibility. However, two previous studies have reported the initial PZA resistance to be 6% and 8%, respectively [18,23]. In this study, PZA susceptibility testing by BACTEC MGIT 960 PZA revealed 34.6% (52/150) PZA resistance. More specifically, PZA resistance was found in 6% (3/50) of pan-susceptible isolates and 49% (49/100) of MDR-TB isolates. The results were correlated with those obtained from South Africa indicating 53.3% (68/127) PZA resistance among previously treated TB patients but a lower resistant rate of 2.1% (1/47) in drug susceptible isolates [14].

PZA resistance is usually associated with defects in PZase activity. Several studies attempted to detect enzyme activity and utilised susceptibility testing for PZA [18,19,21,22]. The sensitivity of the PZase assay ranged from 79-96%, whereas the specificity was approximately 98% [20-22]. In this study, PZase activity was detected in all 98 PZA-susceptible *M. tuberculosis *isolates but in only 18 of 52 PZA-resistant isolates. Eighteen isolates with positive PZase activity presented discordant results with the MGIT 960 PZA system, resulting in a sensitivity and specificity of 65.4% and 100% for that assay, respectively. The sensitivity of our PZase assay is low relative to earlier studies. This might be the result of geographic differences among *M. tuberculosis *isolates. Our isolates may have had alternative resistant mechanisms, such as insufficient drug uptake or the presence of an active efflux pump [10], which limits the utility of this test. However, the PZase assay was still useful for screening PZA-resistant *M. tuberculosis *isolates and could be used as an alternative method, particularly for low-income countries where the assay was highly sensitive.

The major mechanism of PZA resistance was associated with mutations of the gene coding for pyrazinamidase, *pncA*, in which mutations were scattered along the coding and promoter regions with high diversity [7]. In this study, mutations were found in 49 isolates, of which 39 were PZA-resistant and 10 were PZA-susceptible. However, 17 isolates (7 PZA-resistant and 10 PZA-susceptible isolates) showed either Ile31Ser or Ile31Thr mutations. Of these, 15 isolates (except 2 PZA-resistant isolates) had PZase activity. Previous studies have demonstrated the catalytic residues of *M. tuberculosis *PZase that comprise the active (Asp-8, Trp-68, Lys-96, Ser-104, Ala-134, Thr-135 and Cys-138) and metal-binding sites (Asp-49, His-51 and His-71) [30-32]. Taken together with our results, the mutation at Ile-31 did not appear to be associated with PZA resistance. Notably, two PZA-resistant isolates harboured the Ile31Ser mutant but possessed no PZase activity. One possible scenario is that these 2 isolates might have PZase activity that is below the limit of detection for the PZase assay.

Twenty-two of 24 mutation types were detected in this study and showed a correlation with PZA resistance (Table 2). Of these, 14 nucleotide substitutions [13,14,29,33-36] and 2 putative promoter region [9,33] mutations were previously reported. There were 6 novel mutation types, consisting of 3 nucleotide substitutions (Leu27Pro, Gly122Ser, and Thr174Ile), 2 nucleotide insertions (G insertion between nucleotide 411 and 412 and GG insertion between nucleotide 520 and 521), and 1 nonsense mutation at Glu127. In agreement with earlier studies, the mutations were diverse and scattered throughout the gene sequence, with the most frequently occurring mutation being His71Asp (8/49 = 16%). This is not surprising, as His71 is located in one of the three preferably mutated regions (positions 3 to 17, 61 to 76, and 132 to 142) [37] and in the metal-binding site. In addition, there were 13 PZA-resistant isolates (25%) with observed PZase activity and no mutations in *pncA*, implying that other unknown mechanisms are involved in PZA resistance.

## Conclusions

This study showed the prevalence of PZA resistance in pan-susceptible and MDR-TB *M. tuberculosis *clinical isolates from Siriraj Hospital, Thailand. MDR-TB isolates had a much higher percentage of PZA resistance (49%) than susceptible isolates (6%). In this study, the sensitivities of the PZase assay and *pncA *sequencing were 65% and 75%, respectively. The results revealed that 25% of PZA-resistant isolates had wild-type *pncA*, indicating that phenotypic susceptibility testing was still necessary. Additionally, some *pncA *mutations are not likely to produce resistance, limiting the use of genotypic methods for PZA susceptibility testing. This information is useful for clinicians in choosing suitable drug regimens for treating TB patients. This study also indicated that the automatic addition of PZA in the treatment regimen of MDR-TB patients would have less benefit in Thailand and would increase the risk of XDR-TB development or render treatment ineffective. Therefore, PZA susceptibility testing in MDR-TB patients should be performed before starting or adjusting treatment regimens.

## Authors' contributions

JJ carried out all experiments and drafted the manuscript. TP conceived of the study, participated in its design, performed data analysis and interpretation and helped to draft the manuscript. ML helped to revise the manuscript. AC conceived of the study, participated in its design, helped to critically revise the manuscript and gave final approval of the manuscript. All authors read and approved the final manuscript.
