# Correction: Surveillance of pyrazinamide susceptibility among multidrug-resistant *Mycobacterium tuberculosis *isolates from Siriraj Hospital, Thailand

**DOI:** 10.1186/1471-2180-10-278

**Published:** 2010-11-09

**Authors:** Jirarut Jonmalung, Therdsak Prammananan, Manoon Leechawengwongs, Angkana Chaiprasert

**Affiliations:** 1 Department of Microbiology, Faculty of Medicine Siriraj Hospital, Mahidol University, Bangkok, 10700, Thailand; 2 National Center for Genetic Engineering and Biotechnology, National Science and Technology, Development Agency, Ministry of Science and Technology, Pathumthani 12120, Thailand; 3 Vichaiyut Hospital, Setsiri Road, Bangkok 10400, Thailand; 4 Drug-Resistant Tuberculosis Research Fund, Siriraj Foundation, Bangkok 10700, Thailand

## Correction

After galley proof of the manuscript, we found three mistakes of the nucleotide positions (G222C, G364A and C520T) and codon numbers (Gly74Arg, Gly122Ser and Thr174Ile) that have to be corrected but it was unable to make any change because the publication of this work is on going [1]. After revision, Table two (Table 1 in this manuscript) and some information in the discussion part were changed. There were only 5 novel mutation types found in this study, consisting of 2 nucleotide substitutions (Leu27Pro and Thr174Ile), 2 nucleotide insertions (G insertion between nucleotide 411 and 412 and GG insertion between nucleotide 520 and 521), and 1 nonsense mutation at Glu127.

**Table 1:** Results of *pncA *gene sequencing of 150 *M. tuberculosis *clinical isolates.

			*pncA *mutation	
			
*M. tuberculosis *strains (no. of isolates)	MGIT 960	PZase assay	Nucleotide change	Amino acid change
Susceptible (46)	S	+	wild-type	no
Susceptible (1)	S	+	T92G	Ile31Ser
Susceptible (2)	R	+	wild-type	wild-type
Susceptible (1)	R	+	T92C	Ile31Thr

MDR-TB (42)	S	+	wild-type	wild-type
MDR-TB (9)	S	+	T92C	Ile31Thr
MDR-TB (34)	R	-	A(-11)G (1)	no
			A(-11)C (1)	no
			T56G (1)	Leu19Arg
			T80C (1)	Leu27Pro
			T92G (2)	Ile31Ser
			T104C (1)	Leu35Pro
			T134C (1)	Val45Ala
			G136T (1)	Ala46Ser
			T199C (1)	Ser67Pro
			C211G (8)	His71Asp
			G215A (1)	Cys72Tyr
			G289A (3)	Gly97Ser
			C312G (2)	Ser104Arg
			G322C (1)	Gly108Arg
			G373T (1)	Val125Phe
			G379T (1)	Glu 127 Stop
			G394A (1)	Gly132Ser
			G insertion b/w 411-412 (1)	
			T416G (1)	Val 139 Gly
			C425T (1)	Thr 142 Met
			G436A (1)	Ala 146 Thr
			GG insertion b/w 520-521 (1)	
			C530T (1)	Thr 177 Ile
MDR-TB (11)	R	+	wild-type	no
MDR-TB (4)	R	+	T92C (3)	Ile31Thr
			T92G (1)	Ile31Ser

We regret any inconvenience that the mistake might have caused. We wish to thank Dr. Claudio Köser, Department of Genetics, University of Cambridge, for bringing this matter to our attention.
